# Flipping Classrooms in a School of Public Health

**DOI:** 10.3389/fpubh.2017.00073

**Published:** 2017-04-12

**Authors:** Steven W. Howard, Darcell P. Scharff, Travis M. Loux

**Affiliations:** ^1^Saint Louis University, St. Louis, MO, USA

**Keywords:** flipped classroom, public health, health management, Council on Education in Public Health, Commission on Accreditation of Healthcare Management Education, accreditation

## Abstract

Alternative course formats are gaining increasing attention in higher education. The literature provides a number of examples and studies of flipped classrooms in the medical sciences and liberal arts and sciences. However, fewer than five papers on flipped classes in graduate public health courses have been published, and none in health management. Because graduate public health education is competency based, it seems that a flipped approach with its applied nature would be an appropriate form of teaching public health courses. This paper describes three successfully flipped courses taught in a school of public health. We provide a rationale for flipping, description of each course, and lessons learned. Once some of the challenges are overcome, we believe flipping courses can provide an alternative approach that enhances active learning in applied, public health, and health management courses.

## Introduction

Alternative formats of course delivery such as online, hybrid, and flipped continue to gain popularity. Definitions of flipped courses vary, but in general, lectures are viewed and supplemental readings are done outside of classroom time to gain basic knowledge. In-class time is reserved for active learning such as activities and assignments (that would traditionally have been homework) (1–6). Flipped courses force students to no longer be passive observers, but active participants in learning (6, 7).

There is increasing evidence about the benefits of the flipped classroom in higher education in undergraduate and graduate education. The flipped approach can stimulate increased student–teacher interactions (5) and promote active learning and problem solving (8, 9). This approach enhances critical thinking skills (10–13) by forcing students to engage with the course material at a deeper level than with traditional classroom approaches, and it can respond to Millennials’ faster paced thinking and information processing (14). Recorded lectures allow students to review the didactic information on their own time (15). Flipped classrooms use time more efficiently as learning occurs through application of the concepts (provided in the recorded lectures and supplemental readings) during class time (16–18).

The current literature provides an abundance of papers that describe or measure the impact and/or satisfaction with flipped courses in higher education. Although our literature search found that flipped classrooms were reported in a variety of health and liberal arts courses, only three articles were written about courses in public health programs or schools of public health, all at the graduate level, and none in graduate health administration (8, 19, 20). Accredited public health and health administration education is competency based, that is, it intentionally prepares students with knowledge, skills, and attitudes appropriate for the jobs for which they are being trained (21, 22). The competencies on which MPH and MHA programs are developed typically follow the guidance of accrediting agencies, such as the Council on Education in Public Health (CEPH) or the Commission on Accreditation of Healthcare Management Education (CAHME) (21). A competency-based approach trains students to be able to adequately function in their profession upon graduation. Students are trained to “do” as opposed to merely “know” or “believe” (21). Since the flipped classroom focuses attention on the application of didactic information presented in recorded lectures and supplemental reading (9, 23), it seems this approach would be useful in preparing students to be competent in their fields of study.

We flipped three types of graduate public health and health management courses (survey/theory, methods, and project based) to allow more in-class application time, given our accredited programs’ directive to enhance student competency. In this paper, we describe the purpose, methods, and evaluation of the courses. We describe the challenges of converting the courses from traditional face-to-face to the flipped format. Finally, we list the lessons learned from our experiences.

## Methods

### Overview of Courses

The primary purpose for flipping the courses was to allow for more application of concepts (theory, statistical tools, and project planning) by spending more classroom time actually applying the material followed by in-depth discussions about the rationale for using the concepts.

The courses described below are taught in a CEPH accredited school of public health, and one in a CAHME accredited program; therefore, the courses are developed based on a set of program competencies (Table S1 in Supplementary Material). The MPH competencies follow CEPH guidance, as well as faculty, alumni, and community partner input. The MHA competencies were developed by faculty in the program, alumni, and community partners and are known to be one of the three most broadly accepted competency models in the health management field (24). Programmatic competencies drive the development of the curriculum. Each course identified associated learning objectives that drive teaching strategies and assessment. We list the competencies, associated learning objectives, teaching strategies, and assessment for each course in Table 1.

**Table 1:** **Description of flipped courses**.

Course number and name	Competencies addressed	Learning objectives	Teaching strategies	Assessments	Mid-course adjustments
BSH5000: Behavioral Science and Public Health (3 credit hours)	Use an ecological framework to explain the role of individual, social, and community factors that affect the health of individuals and populations and identify general guidelines to intervene and evaluate at multiple levels within the framework	Define the purpose of social–ecological models of health promotion and the levels within the framework Differentiate between models and theories that can be used at the individual, social, organizational, and community levels of the ecological framework	Online lectures and associated readings Class activities to identify theories and their operationalization at various levels of the model Class discussion	Theory application assignment Final theory-based intervention	Added a short in-person review lecture of the week’s material; presentations often generated rich discussions that added more variety to the course
	Identify how basic theories, concepts, and models from a range of social and behavioral disciplines used in public health research and practice can be used to inform public health practice	Determine the appropriate application of theory to understand or explain behaviors and develop interventions For each model discussed in class, describe its overriding principle, constructs, and their relationships	Online lectures and associated readings Class activities specific to use of theories in survey and intervention development Class discussion		
BST5000 Principles of Biostatistics (3 credit hours)	Describe the approaches to disease prevention and control using tools from the five core areas of public health: behavioral science, biostatistics, environmental health, epidemiology, and health management and policy	Distinguish between numerical and categorical data, including which methods to use for each Know the appropriate application and limitations of hypothesis tests and regression methods	Online applied lecture videos In-class conceptual lecture Presentations of published public health research with focus on statistical methods Discussions of assigned readings	Multiple choice quizzes re: videos Weekly homework assignments Two applied projects In-class presentation of public health research In-class conceptual midterm and final exams	Added a short in-person review lecture of the week’s material
	Appropriately utilize qualitative and quantitative data in order to effectively address public health problems	Choose an appropriate graphical or tabular display for a given data set and question Determine which basic statistical method(s) is/are most appropriate to analyze the data at hand Analyze data using fundamental statistical methods	Online applied lecture videos Group homework and projects focused on applying statistical methods using statistical software and interpreting results		
	Use an evidence-based approach for the development of public health programs and policies	Draw conclusions from statistical analyses and place them into the appropriate public health context	In-class conceptual lecture Group homework and projects focused on applying statistical methods using statistical software and interpreting results Presentations of published public health research with focus on statistical methods		
HMP5340: Health Care Marketing (3 credit hours)	Select and use competitive and collaborative strategies appropriately Design and implement business plans for health programs and services Provide effective communication linkages within an organization and to its external environments Identify and use appropriate communication strategies based on audience characteristics and communication goals	Define marketing and describe its components, constructs, and theories. Apply each component of marketing. Incorporate web and social marketing approaches into the marketing plan. Explain how marketing relates to the strategic positioning of an organization. Analyze cases to derive recommendations for actions. Communicate effectively in written and oral formats. Work well as a member of a team.	Online lectures and associated readings from textbook, health-care marketing-related articles, and examples of ads Class discussion about ads students have seen, and their thoughts on effectiveness of each. Discussion of other elements of marketing they have observed at work or in their surroundings Q&A sessions Guest speakers: marketing experts from multiple sectors of the health-care industry (hospital, clinic, insurance, medical equipment) Student presentations and discussions re: current status of their marketing plans (at multiple points in the semester) Group cases/activities	Multiple choice and short essay tests re: lectures, readings, and guest speakers Marketing plan group project In-class presentation of group project milestone Presentation of final marketing plan	Added short face-to-face lectures/discussions to integrate text, and online elements

Each course conducted a mid-semester review either by the professor (BSH5000) or by the university teaching center (BST5000 and HMP5430). Such reviews are important to assess the progress of student learning and to inform course-corrections, especially when piloting a pedagogical change like the flipped format. Because recorded lectures are unidirectional, intermittent reviews and feedback sessions help compensate for this, enabling the instructor to make adjustments to better meet students’ needs. Each of the courses required fine-tuning to better meet students’ needs and improve their competency achievement. For example, the midcourse assessment suggested that a short review of the lecture and reading material at the beginning of class would be useful, and therefore was incorporated into each class session.

### Course Faculty

The courses that used the flipped approach were taught by full-time faculty in the College. Dr. Darcell P. Scharff is a behavioral scientist and teaches courses in the Behavioral Science and Health Education Department and in the Maternal Child Health program. Dr. Travis M. Loux teaches courses in Biostatistics at both the undergraduate and graduate levels. Dr. Steven W. Howard teaches courses in the Health Management and Policy Department in the residential and executive MHA programs.

## Results

### BSH5000: Behavioral Science in Public Health

#### Type of Course

This is a required survey course for all MPH students, which introduces concepts, principles, and theories of behavioral science and health education in public health. The course uses a social–ecological framework to organize the six main theories presented in the class. Students study the purposes and constructs of each theory and learn to apply the theories to either understand public health problems or develop interventions. The course introduces students to the importance of community engagement when addressing public health problems as well as program evaluation to determine impact.

#### Traditional Course Format

The traditional course was lecture based. Students prepared by reading the book chapter(s) and/or articles and were required to submit a question or comment on each reading. The questions guided in-class group discussion. On occasion, an exercise related to the course content was used in class, but these were often truncated because of time constraints. Students wrote short reports on theory application and created a theory-based intervention for the final project.

#### Method of Flipping

All lectures were recorded by teaching faculty and made available to students a week prior to class. The expectation was that students watched the recorded lectures, read the accompanying book chapter and articles, and read or watch any additional materials for the week’s lessons. In addition, the students submitted weekly questions/comments (as they did in the traditional format), which then guided the discussion that occurred at the beginning of each class. Lectures addressed one topic, were 30 min or less, and provided an associated PowerPoint presentation. Class time was spent primarily on exercises that helped students learn to apply the course material in real world and public health situations. For example, when studying the transtheoretical model (TTM), students participated in a group exercise to develop survey items and intervention strategies based on the TTM specific to a public health issue. On large flipchart note paper placed around the classroom, they developed a survey question that assessed a stage of change or an intervention strategy specific for the stage. Once completed, the students critiqued their colleagues’ work, which stimulated relevant discussion. To further assess student competency, they were required to present an application of a theory and develop a theory-based intervention for the final project.

#### Course Evaluations

The Individual Development and Educational Assessment (IDEA) course evaluation system used by the college reports a “progress on relevant objectives” score, which is a weighted average of student ratings of the progress they reported on objectives pre-selected by faculty as important or essential (IDEA). The IDEA course evaluation system provides scores that reflect the students’ perceptions of how well they achieved the course objectives, as well as their perceptions of the quality of the course overall, and the instruction (25). Table 2 lists the results of the IDEA scores for each course taught in a traditional and flipped format.

**Table 2:** **Course evaluation scores in traditional and flipped formats**.

	BSH-5000		BST-5000		HMP-5340	
	Traditional	Flipped	Traditional	Flipped	Traditional	Flipped
**Progress on relevant objectives^a^**						
Raw	4.4	4.2	3.9	4.4	4.3	4.3
Adjusted^b^	4.5	4.4	3.8	4.1	4.1	3.8
**Course rating^c^**						
Raw	3.6	4.2	3.8	4.2	3.7	4.1
Adjusted^b^	3.5	4.2	3.7	4.1	3.4	3.6
**Instructor rating^c^**						
Raw	3.7	3.9	4.4	4.5	4.2	4.1
Adjusted^b^	3.6	4.3	4.5	4.4	4.0	3.8

*^a^Scores are weighted averages of student ratings of the progress they reported on learning objectives. The score represents a 5-point Likert scale defined as 1 = no apparent progress; 2 = slight progress; 3 = moderate progress; 4 = substantial progress; and 5 = exceptional progress*.

*^b^Adjusted for student motivation, work habits, effort, size of class, and course difficulty*.

*^c^The average student agreement with statements that measure teacher and course excellence assessed on a 5-point Likert scale where 1 = Definitely false and 5 = Definitely true*.

For the BSH5000 course, scores on “progress toward relevant objectives” decreased slightly when the flipped course was implemented, but remained higher than 4.0 (on a 1–5 scale). Both, overall course and teaching ratings, improved when taught in the flipped format.

Qualitatively, students commented that they appreciated the ability to use the lectures for review and reinforcement. They also believed the application and discussions components significantly enhanced their learning.

### BST5000 Principles of Biostatistics

#### Type of Course

The introductory biostatistics methods course is taken by all students in the first year of their MPH studies. The course introduces students to fundamental topics in applied statistics, such as basic probability and descriptive statistics, *t*-tests, one-way ANOVA, chi-squared test for independence, and McNemar’s test for paired data, correlation, and linear and logistic regression. Within these topics, students learn about conceptual issues such as sampling methods, multiple comparison problems, and interpretation of statistical results.

#### Traditional Course Format

The traditional BST5000 course was heavily lecture based. Meetings generally started with a brief in-class quiz covering material from the prior week. Students would then listen to a lecture for the remaining part of the class, possibly with some in-class activity. Weekly assignments consisted of a mix of approximately 10–15 textbook problems and instructor-created problems derived from the week’s material and were due the following week. Two assignments were more in-depth “labs” for which students were asked to handle larger data sets with more complicated, in-depth questions that required students to synthesize multiple weeks’ worth of content.

#### Method of Flipping

The flipped format includes approximately three lecture videos (accompanied by a PowerPoint presentation) per week to be watched prior to class. Each video ranges from 5 to 15 min, covers the mechanics of a single statistical method, and is followed by a short multiple choice quiz. All videos were created by the teaching faculty. Students read from the required textbook, as well. In class, students receive a brief in-person review tying together the conceptual links among the video lectures. Students then begin working on a weekly assignment or lab similar to that in the traditional course, which they finish as homework outside of class.

#### Course Evaluations

The raw scores improved for all three indicators on the IDEA system, compared to scores for the traditional course taught the previous year (Table 2).

Qualitatively, students reported using the video lectures both as preparation for class and as review material for the course exams and future exams. Students also saw the in-class time working together as beneficial for making connections with other students, learning to use statistical software, and completing class work.

### HMP5340 Healthcare Marketing

#### Type of Course

The healthcare marketing course is a required project-based course taken by MHA students. The class includes a semester-long group project in which students act as junior management consultants to produce a comprehensive strategic marketing plan for a health-care organization client.

#### Traditional Course Format

The course was historically lecture based, with short, infrequent discussions or project work times, and a few guest speakers from industry. Outside of regular class time, students were expected to read the text and complete individual and group homework assignments, including working on the marketing plan project. In class, students simply listened to the lecture, took notes, participated in limited discussions, and took exams.

#### Method of Flipping

Recorded lectures reinforced the concepts from the week’s assigned reading and were 30 minutes or less per recording. All lectures were developed by the primary faculty teaching the course. Students were expected to have read the text and viewed the recorded lectures before each face-to-face class.

After flipping the class, the in-class sessions included summary lectures, with emphasis on discussion to better engage students. Students worked in groups on case studies that exemplify that week’s course content. Students also worked in class on their group projects. During project work sessions, the professor acts in the role of senior consultant and circulates among the teams to answer questions and help them progress. The student teams, playing the role of junior consultants, report to their senior on the progress they have made on their clients’ projects every 14–21 days. During the semester, one such milestone report is given in front of the class, and the others are scheduled at the professor’s office. The course content is aligned with the milestone reviews. At the end of the semester, the groups present their proposals to the class, often with client representatives in the audience. The flipped format also permits additional marketing guest speakers from industry, and now represents all the major subsectors of the health-care industry.

#### Course Evaluations

During the first year, the class was taught in a completely flipped format, the raw scores for “Progress on Relevant Objectives” and student satisfaction with the professor dropped (compared to the last year the class was taught in traditional format). Very few of the qualitative comments students submitted in the IDEA evaluations pertained to the flipped format, but the ones that did were unhappy about the recorded lectures, relatively unstructured in-class sessions, and quantity of work in the class.

The university teaching center provided consultation on improving the course. In-class time was better structured, using Q&A to directly tie the recorded lecture material to students’ group projects. The professor also more clearly structured the milestone checkpoints for the semester-long project, and worked with groups during class time to provide guidance and help them ensure deliverables were met on time.

By the second and third years teaching the class in a fully flipped format, the professor had integrated students’ responses and the teaching center advice from the first year. IDEA scores improved from the first year the course had been flipped and were approaching the levels when the class was taught in the traditional format (Table 2). Qualitatively, student comments indicated that the class format and structure were appealing, interactive, engaging, and realistic, and the flipped format allowed them to go back to lectures as needed.

## Discussion

This paper describes how three public health courses with varying purposes were successfully flipped. The courses were flipped primarily to allow more classroom time for applied activities and learning as a way for students to better gain greater competence. In general, course evaluations indicated that the flipped format was a positive way to help them make progress on the relevant course objectives, i.e., enhance their competence to execute the necessary skills to perform well in their profession. For example, the flipped format gave the biostatistics faculty more time for explaining how to interpret and use data, i.e., to help students hone their ability to turn the numbers into words. Students appeared to demonstrate more complete and critical thinking about how the data can help guide public health solutions. The course changed from a traditional methods course to a solutions-based course. Likewise, the increased in-class time for application in BSH5000 and HMP5340 allowed instructors to assess student learning and make corrections in real time. In BSH5000, this resulted in a number of “ah-ha” moments among students as they came to understand how theory is a useful tool in public health practice. In HMP5340, student competencies were reinforced through more guest speakers providing real-life cases that exemplified the class material. They also made faster progress on their marketing plans and were less frustrated with questions and ambiguities on their projects, since they had in-class time with the professor every week.

This paper was conceptualized after the courses were taught and an evaluation beyond the IDEA evaluation was not planned *a priori*. However, there is extant theoretical framework that may help to explain our observations. Based on work by Bransford and colleagues (26), learning takes place when students understand the information deeply, understand the information in context, apply the information, and organize it for easy retrieval. It is suggested that the effectiveness of flipping is because students can apply knowledge and organize it in such a way to be able to hold it for future use. Flipping allows students to take control of their learning, and thereby deepening its retention (27). More evaluation research on public health courses should be conducted to test this premise.

Flipping courses does not, however, come without challenges. Perhaps, the greatest challenge is increased time both in preparation (2, 9, 28, 29) and during the course. Finding or developing a variety of creative activities that relate to the competencies, while keeping students engaged and productive, requires a significant time investment (29). It is also time-consuming for faculty to master the university’s learning management system, necessary for flipping a course. Balancing the amount and quality of the recording, while addressing the students’ learning needs is often a new skill that requires additional time (30). In addition, flipped courses can result in increased faculty time outside of class with students who do not comprehend the online lectures.

We learned several important lessons related to flipping the courses and provide practical applications related to them below.

- Teaching scores may be impacted both positively and negatively by flipping classes. Although the “progress on objectives” score slightly declined in the theory course, the score remained above 4 or a 5-point Likert scale, which indicates that students perceived substantial progress toward meeting the course objectives. The “overall course” and “instructor” ratings improved in the flipped format for the theory course. We believe this was due in part to the use of brief, to-the-point recorded lectures. Students preferred this approach versus the traditional, hours-long lectures that could frequently stray from the main points. All three metrics improved in the biostatistics courses. The HMP5340 scores were improved after working with the university teaching center, suggesting that careful transition planning is important when considering flipping courses. It is not enough to simply convert your lectures to an online format (31, 32).
- There are numerous resources available to faculty for identifying creative activities to use during the classroom time. Most large universities provide a teaching center that can be an excellent first step in the transition process. Center staff can provide information and training in specific teaching techniques and modalities that are appropriate for the flipped classroom. Working with the center can also reduce preparation time for faculty. Those without centers can find a number of online resources that will be useful for a conversion. Our experience with our teaching center proved to be an important resource to enhance the courses.
- Faculty should allow ample time for the preparation and conduct of flipped courses (2). Lectures need to be re-written so that they are short, relevant, and stay on point. In class activities must be relevant to the recorded lecture and other assigned materials and keep students engaged. In addition, faculty should budget additional office hours (or engage a teaching assistant) to meet with students who may struggle with the more unidirectional recorded lecture approach. However, our experience indicated very few students struggled with the flipped format, especially once we modified the in-class sessions to provide a short summary lecture/discussion in the beginning of the class.
- Faculty should consider using the tracking tool on their learning management systems to track student preparation, particularly after receiving and acting upon student feedback. With less face-to-face feedback, it is more challenging to ascertain which lectures and assignments are resonating with students. By using this tool, faculty are able to understand and address students’ compliance to assignments in preparation for the in-class portion of the course. By addressing these issues early, the faculty can incorporate incentives for students to prepare and participate. For example, by using the tracking function, we learned that students in one class were relying more on the brief face-to-face lectures at the beginning of each class (that were instigated after the mid-course reviews) rather than the recorded lecture and assignments. Subsequently, the short face-to-face lectures were modified to question and answer sessions.
- Some students will be dissatisfied with a flipped approach (9, 33, 34). The increased class time devoted to application, however, can help students see its impact on building their competency. When they realize the flipped format can better prepare them for future courses and/or jobs, it helps them connect the flipped class approach and their learning.

There are limitations inherent in this research pilot. Because we did not set out to evaluate the flipped approach in these courses, we did not plan for a way to systematically measure its impact. Rigorous evaluation studies should be conducted to determine effectiveness by types (survey, methods) and levels (introductory, advanced) of courses taught in schools of public health as a way to add to the evidence for its use. By doing so, we can improve our understanding of when the flipped approach is most effective and, therefore, provide guidance for generalizability. Additionally, the educational settings and/or learning objectives related to the three courses described here may limit the generalizability of our findings in other types of courses.

Flipped classrooms are likely to become increasingly common as we learn more ways to increase their effectiveness and the efficiency of our teaching. Public health and health management programs base their curricula on the development of student competencies, and the flipped format can be a useful tool for competency building in a variety of course types. We believe the flipped approach can be constructive in helping students achieve success in the public health and health management professional fields through developing competencies that will enhance their job performance early in their careers.

## Author Contributions

SH, DS, and TL collaboratively conceptualized this manuscript, and each wrote the section specifically pertaining to his/her own experiences with the flipped class format. DS conducted the majority of the literature review. SH and DS wrote the Introduction, Methods, and Discussion. All participated actively on the revise and resubmit.

## Conflict of Interest Statement

The authors declare that the research was conducted in the absence of any commercial or financial relationships that could be construed as a potential conflict of interest.
