# Supplementary material for: Flipping Classrooms in a School of Public Health
Source: Front Public Health. 2017 Apr 12;5:73. doi: 10.3389/fpubh.2017.00073 (PMC5388680; doi:10.3389/fpubh.2017.00073)
Supplement: Supplementary file 1 [file Table_1.PDF]

Supplemental Table 1 Competencies Required for the MPH and MHA

Core MPH and Concentration Competencies

MPH 1: Explain the organizational structure, financing, politics, and history of the public health and medical care systems.

MPH 2: Recognize the ecological nature of determinants of health that include biological, behavioral, social, environmental, economic, and political factors.

MPH 3: Describe the approaches to disease prevention and control using tools from the five core areas of public health: behavioral science, biostatistics, environmental health, epidemiology, and health management and policy.

MPH 4: Participate in multidisciplinary partnerships and coalitions as both a leader and participant.

MPH 5: Appropriately utilize qualitative and quantitative data in order to effectively address public health problems.

MPH 6: Use an evidence-based approach for the development of public health programs and policies.

MPH 7: Utilize appropriate communication strategies to educate, disseminate, and advocate for health services and preventive interventions.

MPH 8: Apply principles of management in program, organizational, and community initiatives.

MPH 9: Apply principles of ethical reasoning, human rights, and cultural competence when working with and in organizations and communities.

BSHE 1: Apply conceptual models and theories at multiple ecological levels (intrapersonal, interpersonal, institutional, community and policy).

BSHE 2: Examine risk factors and determinants of specific health threats at multiple ecological levels.

BSHE 3: Assess needs, assets, resources and capacity for social and behavioral science interventions at multiple levels (intrapersonal, interpersonal, institutional, community and policy).

BSHE 4: Plan theory and evidence-based interventions (program/policy/environmental change) to improve health.

BSHE 5: Collaborate to implement public health related programs, policies, and environmental changes.

BSHE 6: Develop programs, policies and environmental strategies that address social determinants.

BSHE 7: Conduct evaluations (process, impact, and outcome) of public health related interventions using multiple methods.

BSDP 1: Analyze qualitative and quantitative data to accurately identify biological and other health hazards and measure risks, using epidemiological, statistical, and risk assessment methods and tools

BSDP 2: Use an evidence-based approach to develop and analyze effective human, animal, and environmental hazard control strategies, programs, and policies, taking into account legal and ethical considerations

BSDP 3: Explain the scientific characteristics, including transmission routes and control measures, of major biological hazards that result in human and animal health risk

BSDP 4: Create and disseminate tailored messages regarding biosecurity hazards and risks to responders, the public, the media, and policy makers

BSDP 5: Apply management principles in program, organizational, and community initiatives

BST 1: Articulate the principles of biostatistics.

BST 2: Compute descriptive and multivariate statistical tests and measures of association using statistical software, e.g., SPSS, Excel, SAS & R.
BST 3: Interpret results of statistical analyses.
BST 4: Communicate results of statistical analyses.
BST 5: Follow ethical norms and rules for acquiring, managing, sharing, securing and analyzing data.
EOH 1: Explain the reciprocal relationship between the condition of the environment and the health of its inhabitants.
EOH 2: Identify and describe human hazards in terms of physical, chemical, or biological properties and the potential health consequences of human exposure.
EOH 3: Measure and quantify exposure to environmental and occupational agents and determine associated health risks of exposure.
EOH 4: Suggest mechanisms to control exposure and mitigate or manage risk (engineering, behavioral, policy, etc.).
EOH 5: Assess the impact of environmental and occupational agents on populations and explain how that information is used to establish laws, regulations, and policies.
EOH 6: Communicate technical concepts, findings, and proposals to the public and to other health professionals.
EPI 1: Develop appropriate study designs and analytical strategies to test epidemiologic hypotheses.
EPI 2: Accurately interpret epidemiologic data.
EPI 3: Appropriately communicate epidemiologic findings.
EPI 4: Use data to describe the health of populations.
EPI 5: Critically analyze the epidemiologic literature.
GLOH 1: Assess major forces that influence the health of vulnerable populations from a global perspective
GLOH 2: Prioritize health programs for communicable and non-communicable diseases in low- and middle-income countries with limited resources.
GLOH 3: Utilize a multidisciplinary approach to evaluate programs in low- and middle-income countries, and in poor communities in the U.S., that provide health programs.
GLOH 4: Respond to urgent health problems in low- and middle-income countries, including humanitarian crises and epidemics.
GLOH 5: Plan programs, policies and/or specific interventions to improve health services, or health status of individuals and communities.
GLOH 6: Effectively utilize the tools and channels of diplomacy to implement health programs across cultures, and across borders.
GLOH 7: Utilize appropriate cultural competency and communication skills via a mentored global health practice experience in a developing country.
HMP 1: Leadership: Generate responses to problems, proposals, and politics in health policy and management.
HMP 2: Critical Thinking: Create policy approaches and alternatives within the contextual environment of health services delivery and public health.
HMP 3: Science and Analysis: Effectively use data and appropriate analytical methods to analyze and evaluate health politics, problems and proposals.
HMP 4: Management: Effectively work with people and develop teams to achieve goals.

HMP 5: Political and Community Development: Analyze political feasibility and impacts on key stakeholders to build community and collaborative partnerships at multiple levels (federal, state and local).
HMP 6: Communication: Effectively communicate the health politics, problems and proposals at multiple levels (federal, state and local) and to diverse, relevant stakeholders within and across organizations and communities.
MCH 1: Describe the history and characteristics of MCH population health.
MCH 2: Communicate clearly to a variety of audiences, e.g., professional, lay, from various cultural and ethnic groups.
MCH 3: Use data to identify issues related to the health status of an MCH population.
MCH 4: Develop cultural sensitivity.
MCH 5: Plan and evaluate MCH policies and programs.
PRFP 1: Use an evidence-based approach to develop and analyze effective human, animal, and environmental hazard control strategies, programs and policies, taking into account legal and ethical considerations.
PRFP 2: Explain the scientific characteristics, including transmission routes and control measures, of major biological hazards that result in human and animal health risk.
PRFP 3: Generate responses to problems, proposals, and politics in health policy and management.
PRFP 4: Create policy approaches and alternatives within the contextual environment of health services delivery and public health.
MSPH 1: Identify gaps in current scientific knowledge and develop appropriate research questions.
MSPH 2: Apply appropriate methods of study design to address research questions.
MSPH 3: Apply ethical principles in the conduct of research on human subjects.
MSPH 4: Conduct and interpret basic descriptive and inferential statistics.
MSPH 5: Produce scholarly reports of research and/or programmatic findings.
BSHE 1: Apply conceptual models and theories at multiple ecological levels (intrapersonal, interpersonal, institutional, community and policy).
BSHE 2. Examine risk factors and determinants of specific health threats at multiple ecological levels.
BSHE 3. Assess needs, assets, resources and capacity for social and behavioral science interventions at multiple levels (intrapersonal, interpersonal, institutional, community and policy).
BSHE 4. Plan theory and evidence-based interventions (program/policy/environmental change) to improve health.
BSHE 5. Develop programs, policies and environmental strategies that address social determinants.
BSHE 6. Conduct evaluations (process, impact, and outcome) of public health related interventions using multiple methods.
EPI 1: Develop appropriate study designs and analytical strategies to test epidemiologic hypotheses.
EPI 2: Accurately interpret epidemiologic data.
EPI 3. Appropriately communicate epidemiologic findings.
EPI 4. Use data to describe the health of populations.
EPI 5. Critically analyze the epidemiologic literature.

MHA Competencies (by Domain)

Domain 1: Leadership

1. Articulate, demonstrate and evaluate professional values and ethics.
2. Describe and demonstrate stewardship of organizational resources.
3. Identify, demonstrate and evaluate cultural sensitivity and competence.
4. Identify and articulate community values and needs.
5. Use system and strategic thinking models and methods to make decisions and solve problems.
6. Facilitate and influence the development, implementation and ownership of vision, mission, goals, and strategic objectives and plans.
7. Describe and apply models and methods of organizational change and innovation.
8. Use creative flexible problem-solving methods.
9. Select and use competitive and collaborative strategies appropriately.
10. Identify, explain and resolve critical tension.
11. Identify the bases of power and the political implications and effects of decisions.
12. Identify, explain and utilize effective motivational strategies to elicit desired behavior and inspire others toward a shared vision.
13. Work effectively with other professionals.
14. Identify, value and use team development methods.
15. Demonstrate effective listening and communication skills.
16. Identify effective techniques for working with Boards and governance structures.
17. Demonstrate the ability to make choices.
18. Commit to the execution of organizational strategy.
19. Selects the organizational structure that is appropriate for system functions.

Domain 2: Critical Thinking

1. Able to identify and implement systems and strategic thinking:

Identify the subsystems of the health system broadly defined; their relationships and interactions

Describe the roles, functions and responsibilities of system components

Identify internal/external system structures and social, cultural, economic, financial and political issues/factors

Apply models to develop structures and systems to support team functions

2. Ask the right questions when making decisions.
3. Comprehend cause-and-effect relationships.
4. Evaluate issues from different perspective.
5. Create innovative strategies.
6. Have confidence in the ability to make decisions in the presence of uncertainty.
7. Identify and prioritize managerial alternatives.
8. Able to learn from mistakes.
9. Project future scenarios.

Domain 3: Science/Analysis

1. Identify the scientific method and how to apply it to managerial decision-making and problem solving.
2. Identify and describe the structure and conduct of quantitative analytical methods: economic and financial evaluation, survey research, linear regression analysis, forecasting methods, linear programming, project management and bivariate comparison methods.
3. Identify and describe the structure and conduct of selected qualitative methods: focus groups, case studies, participant observation and ethnographies.
4. Examine data both qualitatively and quantitatively to determine patterns and trends.
5. Evaluate testable hypotheses commonly arising in a management setting, selecting and applying the appropriate quantitative methods.
6. Monitor data and identify deviations, whether for internal processes and procedures or for external trends.
7. Use and manage relevant computer technology (e.g. application software and database technology).
8. Be an educated consumer of more complex analytical methods.
9. Identify and describe information systems and knowledge management.

Domain 4: Management

1. Identify, describe and apply general management concepts regarding the organization of work, economics, finance and decision theory.
2. Work with and through people to achieve organizational goals.
3. Identify, describe and apply basic concepts and tools that are integral to strategic thinking, planning and management.
4. Describe and apply the basic principles and tools of effective human resource management, change management and organizational development.
5. Describe and apply the basic principles and tools of continuous quality improvement concepts and skills to improve work processes.
6. Design and implement business plans for health programs and services.
7. Identify, monitor and interpret the impacts of legal, regulatory and political environments on an organization.
8. Evaluate and modify appropriate governance structures and processes.
9. Identify and address needs of major stakeholders.
10. Provide effective communication linkages within an organization and to its external environments.
11. Measure, track and respond to the changing needs of the customer.
12. Describe and apply the basic aspects of management engineering and the redesign of organizations, systems and processes.

Domain 5: Political and Community Development

1. Identify the major stakeholders of an organization and their specific interests and historical relationships.
2. Effectively solicit and use input from the community in the process of policy and program development and evaluation.
3. Build appropriate collaborations/partnerships with stakeholders at the local, state and national level to create broader influence.

4. Use negotiation, consensus and conflict resolution methods to assist community development and evaluation.
5. Demonstrate “emotional intelligence” or the social and human relationship skills needed to address the diverse needs of the various community stakeholders.

Domain 6: Communication
1. Develop, organize, synthesize and articulate ideas and information.
2. Listen, hear and respond effectively to the ideas and thoughts of others.
3. Speak clearly and effectively before individuals and groups, in formal and informal settings.
4. Write clearly and effectively.
5. Identify and use appropriate communication strategies based on audience characteristics and communication goals.
6. Recognize and use non-verbal forms of communication.
